# Spatial and Temporal Characteristics of the 2009 A/H1N1 Influenza Pandemic in Peru

**DOI:** 10.1371/journal.pone.0021287

**Published:** 2011-06-21

**Authors:** Gerardo Chowell, Cécile Viboud, Cesar V. Munayco, Jorge Gómez, Lone Simonsen, Mark A. Miller, James Tamerius, Victor Fiestas, Eric S. Halsey, Victor A. Laguna-Torres

**Affiliations:** 1 Mathematical, Computational & Modeling Sciences Center, School of Human Evolution and Social Change, Arizona State University, Tempe, Arizona, United States of America; 2 Division of Epidemiology and Population Studies, Fogarty International Center, National Institutes of Health, Bethesda, Maryland, United States of America; 3 Dirección General de Epidemiología, Perú Ministerio de Salud, Lima, Perú; 4 Department of Global Health, School of Public Health and Health Services, George Washington University, Washington, D.C., United States of America; 5 School of Geography and Development, University of Arizona, Tucson, Arizona, United States of America; 6 Instituto Nacional de Salud, Lima, Perú; 7 U. S. Naval Medical Research Unit 6, Lima, Perú; Center for Complex Networks and Systems Research, Indiana University at Bloomington, United States of America

## Abstract

**Background:**

Highly refined surveillance data on the 2009 A/H1N1 influenza pandemic are crucial to quantify the spatial and temporal characteristics of the pandemic. There is little information about the spatial-temporal dynamics of pandemic influenza in South America. Here we provide a quantitative description of the age-specific morbidity pandemic patterns across administrative areas of Peru.

**Methods:**

We used daily cases of influenza-like-illness, tests for A/H1N1 influenza virus infections, and laboratory-confirmed A/H1N1 influenza cases reported to the epidemiological surveillance system of Peru's Ministry of Health from May 1 to December 31, 2009. We analyzed the geographic spread of the pandemic waves and their association with the winter school vacation period, demographic factors, and absolute humidity. We also estimated the reproduction number and quantified the association between the winter school vacation period and the age distribution of cases.

**Results:**

The national pandemic curve revealed a bimodal winter pandemic wave, with the first peak limited to school age children in the Lima metropolitan area, and the second peak more geographically widespread. The reproduction number was estimated at 1.6–2.2 for the Lima metropolitan area and 1.3–1.5 in the rest of Peru. We found a significant association between the timing of the school vacation period and changes in the age distribution of cases, while earlier pandemic onset was correlated with large population size. By contrast there was no association between pandemic dynamics and absolute humidity.

**Conclusions:**

Our results indicate substantial spatial variation in pandemic patterns across Peru, with two pandemic waves of varying timing and impact by age and region. Moreover, the Peru data suggest a hierarchical transmission pattern of pandemic influenza A/H1N1 driven by large population centers. The higher reproduction number of the first pandemic wave could be explained by high contact rates among school-age children, the age group most affected during this early wave.

## Introduction

Although a few quantitative studies have started to shed light on the spatial, temporal, and age specific patterns of mortality and transmissibility levels of historical pandemic events [1], relatively little is known about the spatial-temporal patterns of the 2009 A/H1N1 influenza pandemic at different spatial scales. For instance, historical influenza pandemics have been characterized by a disproportionate impact on morbidity and mortality rates among young individuals, a feature that is in stark contrast with seasonal influenza epidemics [2]. Also, influenza pandemics have been found to disseminate in multiple waves occurring over short time periods. Furthermore, out-of-season influenza activity has been documented in spring and summer months in temperate countries during the 1918 influenza pandemic [3], [4], [5] and the recent 2009 influenza pandemic [6], [7].

The 2009 A/H1N1 influenza pandemic represents a unique opportunity to increase our understanding of the spatial-temporal diffusion patterns of pandemic influenza at different spatial scales, which is crucial for improving public health interventions against future influenza pandemics. In particular, the role of school closure and environmental forcing on the transmission dynamics of pandemic influenza remains debated [8], [9] [10], [11], [12]. Peru is a particularly interesting case study in this respect as it covers a variety of climatic zones associated with diverse influenza seasonal patterns [13] and public health authorities moved the national winter school vacation period by 2-weeks, in an attempt to mitigate the impact of the 2009 A/H1N1 pandemic. Whether the winter school vacation period had any impact on the transmission dynamics of the pandemic in Peru has yet to be evaluated, and this could provide insight into the role of school closures on control of future pandemics [8], [9], [14], [15], [16]. Here we analyze the spatial patterns of age-specific time series of influenza-like-illness (ILI) and laboratory confirmed A/H1N1 influenza cases collected in Peru during 2009. We also quantify the association between local pandemic patterns, absolute humidity, and demographic factors.

## Materials and Methods

Peru is a South American country sharing borders with Bolivia, Brazil, Chile, Colombia, and Ecuador, with a heterogeneously-distributed population of 28 million (average population density ∼22 per km^2^). The country is divided by the Andes Mountains in naturally distinct regions (highlands, coastal desert and jungle). All regions span the entire length of the country from latitude 3°S to latitude 18°S, and experience different influenza seasonal patterns in inter-pandemic years [13]. Perú is divided into 24 administrative departments composed of 196 provinces ranging in population size from 7000 to about 7.5 million people [17].

We relied on the epidemiological and virological surveillance network for influenza and other respiratory viruses conducted since 1998 by the Peruvian Ministry of Health (MoH) [18]. In 2006, the MoH of Peru expanded the surveillance network by joining efforts with the US Naval Medical Research Unit – 6 (NAMRU-6) and scaled up its coverage to a total of 50 inpatients and outpatients Sentinel health care centers, uniformly distributed across geographic regions of Peru [13], [19]. Moreover, as a result of the global pandemic alert issued by the World Health Organization (WHO) on May 9, 2009, the MoH of Peru intensified surveillance efforts through a public health directive [20] and expanded the identification of influenza-like-illnesses (ILI) to include all public and private health care centers in the country. ILI was defined as a sudden onset of fever (≥38°C) and cough or sore throat fewer than five days in duration, with or without general symptoms such as myalgias, prostration, headache, or malaise [21]. Nasal and/or oropharyngeal swabs were taken from a random sample of ILI patients and sent to the Instituto Nacional de Salud and NAMRU-6 for testing by reverse transcriptase polymerase chain reaction (RT-PCR) [18]. Case definitions and laboratory diagnostics did not change throughout the pandemic period covered in our study. However on July 7, 2009, public health authorities ordered prioritization of hospitalized ILI cases for A/H1N1 influenza testing in all health care centers, except for the 50 official sentinel units where testing of mild and severe ILI cases was maintained [22]. A chronology of events relating to the 2009 A/H1N1 influenza pandemic in Peru is given in Table 1.

**Table 1:** Timeline of events relevant to the detection, control, and suspension of school activities during the 2009 influenza pandemic in Peru.

Dates	Events
April 29, 2009	The World Health Organization raises the pandemic alert to level 5 after successful dissemination of the pandemic virus to several countries
April 30, 2009	Travel restrictions to Mexico are put in place
May 9, 2009	First confirmed case with pandemic A/H1N1 influenza is diagnosed in Peru and the Ministry of Health issues a public health directive to expand the identification of influenza-like-illness (ILI) across all public and private health care centers in the country [20].
July 16–Aug 6, 2009	Winter student vacation period
July 7, 2009	Practice of testing focuses on hospitalized cases in all health care centers except for the 50 official sentinel units where testing of mild and severe cases was maintained [22].

We obtained patient age (years), date of symptoms onset, and reporting department (n = 24) and province (n = 134) for ILI and laboratory-confirmed influenza A/H1N1 cases reported between May 1 and December 31, 2009. We also obtained 2009 population size and density (people/km^2^) estimates for Peru's provinces and departments, using the National Institute of Statistics and Informatics (http://proyectos.inei.gob.pe/mapas/bid).

### Spatial distribution of A/H1N1 influenza

**Figure 1:**
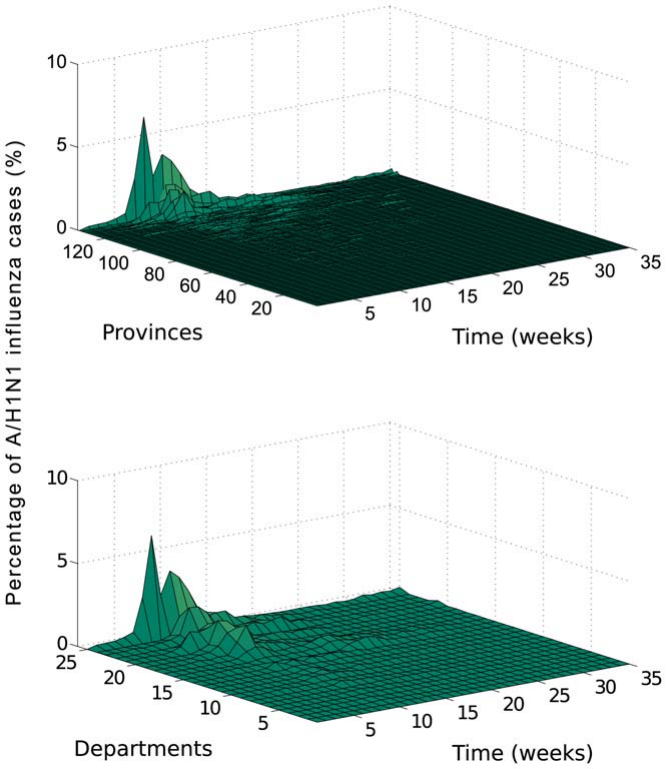
Weekly fraction of A/H1N1 influenza cases (%) during the 2009 A/H1N1 influenza pandemic in Peru at two different spatial scales: (a) 134 provinces and (b) 24 departments, May 1 to December 31, 2009.

We compiled age-specific time series of ILI and A/H1N1 influenza cases by day of symptom onset for May–December 2009 at the refined spatial scale of provinces (N = 134), as well as the coarser scale of departments (N = 24) (Figure 1). For each spatial unit we recorded the cumulative number of cases and peak day, defined as the day with the maximum number of new cases. We also estimated the day of pandemic onset defined as the first day of the period of monotonously increasing cases leading up to the peak of A/H1N1 cases, as in [23]. We then investigated geographic variation in the timing of pandemic onset across departments and provinces and their association with demographic factors and distance from Lima.

### Geographical variation in pandemic timing and absolute humidity trends

Absolute humidity was shown to affect influenza survival and transmission in previous studies [24], and was linked to the timing of onset of seasonal and pandemic influenza outbreaks in the US [11], [12]. Here we explored the relationship between daily variation in absolute humidity and the temporal profile of the pandemic in the 134 provinces of Peru. For this purpose, we compared daily variation in number of new A/H1N1 influenza cases and average specific humidity in Peru weighted by the total number of A/H1N1 cases reported in each province from May 1 to December 31, 2009. Specific humidity (g/kg) is a proxy for absolute humidity and was calculated from daily averages of temperature, relative humidity, and surface pressure obtained from the National Center for Environmental Prediction-National Center for Atmospheric Research (NCEP-NCAR) global reanalysis [25].

### Estimation of the reproduction number

We estimated the reproduction number, R, using a simple method that relies on the estimation of the growth rate, “*r,*” by fitting an exponential function to the early ascending phase of the epidemic curves in Lima and the rest of Peru [5], [23], [26], [27]. The early ascending phase was determined as the period between the day of pandemic onset (as defined earlier) and the midpoint between the onset and peak days. The reproduction number was calculated by substituting the estimate for *r* into an expression derived from the linearization of the classical Susceptible-Exposed-Infectious-Recovered (SEIR) transmission model [26], [28]:
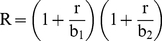
(1)where 1/b_1_ and 1/b_2_ are respectively the mean latent and infectious periods which are assumed to be exponentially distributed. Hence, the mean generation interval between two successive cases is given by 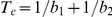. As a sensitivity analysis, we also obtained an upper bound estimate for the extreme case of a fixed generation interval (delta distribution), using the following expression [26]:
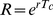
(2)We assumed a mean generation interval of three (1/b_1_ = 1.5 days and 1/b_2_ = 1.5 days) and four days (1/b_1_ = 2 days and 1/b_2_ = 2 days), which are within the range of mean estimates for the 2009 influenza pandemic [29], [30], [31], [32]. We assessed the sensitivity of our estimates to small variations in the definition of the ascending phase used to estimate the exponential growth rate (+/−4 days).

### The impact of the winter school vacation on the age distribution of cases and pandemic burden

We used two complementary approaches to assess the association between the transmission dynamics of the pandemic in Peru and the 2-week winter student vacation period, which started after the peak of the pandemic on July 16 and ended on August 6, 2009. First, we evaluated weekly trends in the ratio of incident A/H1N1 cases among the student population (ages 5–20 years) to incident cases among all other age groups in the Lima metropolitan area and the rest of Peru. A decline in the ratio of school-age to other cases is suggestive of an impact of school closure on transmission of pandemic influenza. However this approach does not take into account the natural dynamics of the pandemic and rapid depletion of susceptibles occurring in the first weeks of the outbreak, especially among highly connected individuals such as school-age children. To take into account susceptible depletion, we used a second approach based on an age-structured mathematical model of influenza transmission, tailored to the epidemiology of pandemic influenza and the population of Peru (Text S1). We quantified the expected reduction in the final pandemic attack rate as a function of the reduction of the transmission rate among the student population associated with school closing, the timing of the school closing period in relation to the pandemic peak, and R_0_ values in the range 1.6–1.8 [29].

## Results

### Trends in testing and influenza-positivity rates

A total of 22,994 ILI cases were reported to the Peruvian Ministry of Health from May 1 to December 31, 2009. Of all reported ILI cases, 18,139 were laboratory tested for A/H1N1 influenza infection (78.9%), and 8,994 cases were confirmed as having A/H1N1 influenza (39.1%). The average RT-PCR positivity throughout the pandemic was 49.6% (see also Figure A in Text S1 for time trends in RT-PCR positivity rate). Daily timeseries of ILI and A/H1N1 influenza cases in the greater Lima metropolitan area and the rest of Peru were highly synchronized (Spearman rho = 0.8–0.9, P<0.0001).

Overall testing rates were higher in the greater Lima metropolitan area (86%) than in the rest of Peru (56%), and rates remained relatively stable over the entire pandemic period with an average weekly testing rate of 73.1% (95% CI: 72.6, 73.7). There was an initial increase in average testing rates nationally from 60 to 80% during the first 6–7 weeks of the pandemic (Figure B in Text S1), a peak testing rate of ∼85% during the period of highest incident cases and then a gradual decline until it reached 60% on week 23 of the pandemic. There were no substantial differences in testing rates by age or geographic regions (Figure C in Text S1).

### General description of the pandemic profile in Peru

**Figure 2:**
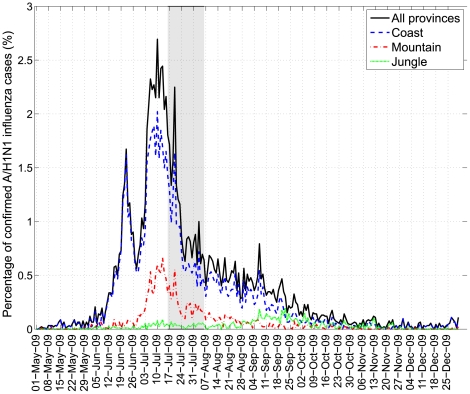
The daily evolution of the A/H1N1 influenza cases (% of total cases during the study period) in coastal, mountain, and jungle regions of Peru, May 01, 2009 to December 31, 2009. The shaded area corresponds to the school closure period (07/16–08/06).

**Figure 3:**
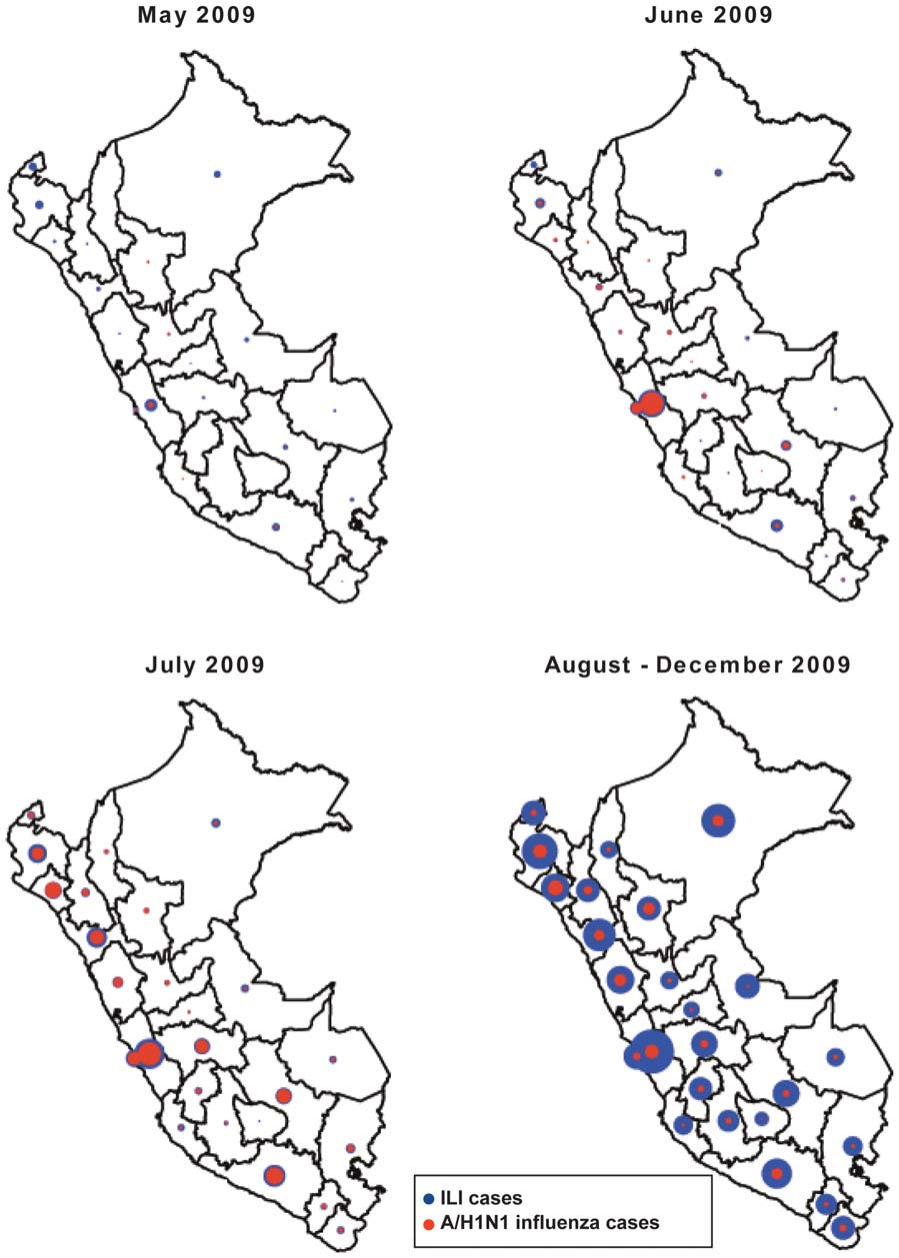
The distribution of ILI and A/H1N1 cases across the 24 Peruvian departments for the months of May, June, July, and August–December, 2009. Pandemic A/H1N1 influenza cases first focused on the Lima metropolitan region and then disseminated to other areas of the country in subsequent months. Circle size is proportional to the number of cases in each time period.

A total of 134 provinces reported A/H1N1 influenza cases during the pandemic period, May–Dec, 2009. Only 50 provinces reported >50 A/H1N1 cases during the pandemic period and most cases were reported in the most populous departments of Lima and Callao (34%). The regional distribution of A/H1N1 influenza cases in Peru reveals that a first pandemic wave mainly affected the coastal region and peaked on June 22, 2009. A second more widespread wave peaked in mid-July, 2009 and was synchronized in coastal, mountain, and jungle regions. The jungle region experienced a resurgence of A/H1N1 influenza later in the year, as the daily curve of new cases peaked on September 22, 2009 in this region (see Figure 2 for regional time series of A/H1N1 cases and Figure 3 for a map).

**Figure 4:**
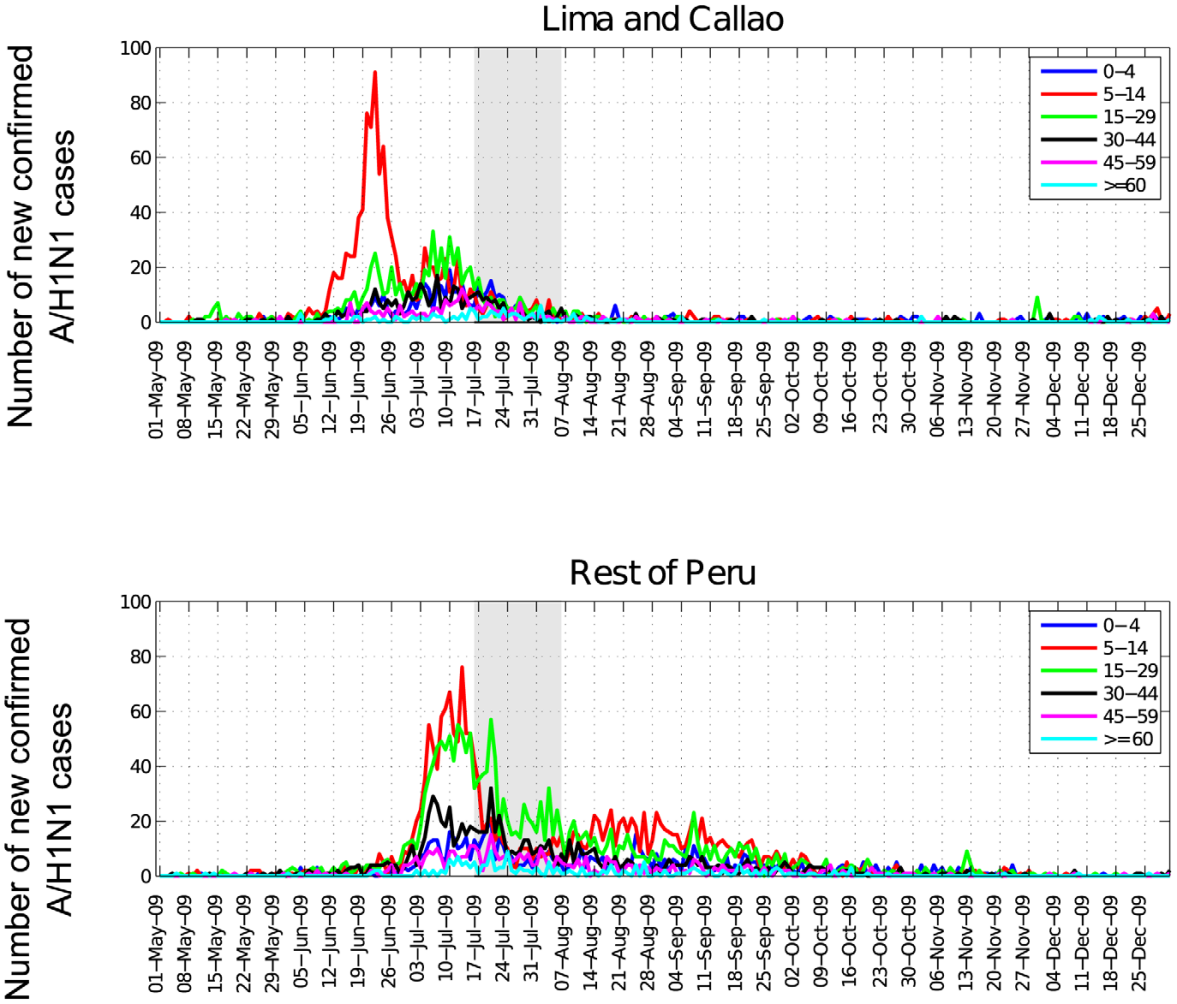
The daily evolution of the pandemic A/H1N1 influenza incidence according to age groups in Lima and Callao and the rest of the country, May 1, 2009 to December 31, 2009. The shaded area corresponds to the 2-week winter school break (July16^th^ to August 6^th^).

The first wave of the pandemic was concentrated in school-age children in the greater Lima metropolitan area and then disseminated across all age groups (Figure 4). Overall, the median age of A/H1N1 influenza cases was 16 years (range: 0 to 98 years). The age distributions of cases was lower in the Lima metropolitan area than in the rest of Peru with a median age of 14 and 17 years, respectively (Wilcoxon test, P<0.009).

### Spatial-temporal variation of the timing of the pandemic onset

Because timing of pandemic onset was geographically asynchronous in Peru (Figure 1), we explored the association between pandemic onset, demographic factors, and absolute humidity in the 134 provinces. At the province level, the timing of the pandemic onset was moderately and significantly associated with population density (Spearman rho = −0.44, P = 0.03; Figure D in Text S1). There was no significant association between the timing of the pandemic and population size (rho = −0.25, P = 0.23) or distance from Lima City (rho = 0.31, P = 0.13). Moreover, timing of pandemic onset at the province level was not associated with a decline in absolute humidity levels within 30 days of onset (Spearman rho = 0.16–0.30, P>0.14, Figure E in Text S1).

At the department level, timing of pandemic onset was moderately and significantly correlated with population size (rho = −0.51, P = 0.03) and population density (rho = −0.49, P = 0.03).

### Trends in reproduction number across pandemic waves

Estimates of the reproduction number and their corresponding confidence intervals were obtained for the greater Lima metropolitan area and the rest of Peru (Table 2 and Figure F in Text S1). Using a mean generation interval of three days and assuming exponentially-distributed latent and infectious periods, the mean reproduction number was estimated to be 1.7 (95% CI: 1.6–1.7) for the greater Lima metropolitan area and 1.3 (1.3–1.3) for the rest of Peru. An upper bound of the reproduction number is also provided in Table 2, in the extreme case of a fixed generation interval of 4 days, suggesting that R remained below 2.2 throughout the pandemic in Peru. There was no significant trend in testing rates during the exponential phase of the pandemic from which the growth rate was measured (P>0.45). R estimates varied little when the time period selected to estimate the growth rate increased or decreased by four days (difference of 0.1–0.2 for the R estimates for the greater Lima metropolitan area and less than 0.05 for the R estimate for the rest of Peru).

**Table 2:** Mean estimates of the reproduction number and corresponding 95% confidence intervals in the greater Lima metropolitan area and the rest of Peru.

Pandemic wave	Geographic region			
	Lima and Callao		Rest of Peru	
	3-day serial interval	4-day serial interval	3-day serial interval	4-day serial interval
**Exponential latent and infectious periods**	1.65 (1.63, 1.67)	1.90 (1.87, 1.93)	1.31 (1.29, 1.32)	1.42 (1.41, 1.43)
**Fixed generation interval**	1.76 (1.74, 1.80)	2.14 (2.09, 2.18)	1.33 (1.32, 1.34)	1.47 (1.45, 1.48)

The serial interval is assumed to be exponentially distributed or fixed, with a mean of three or four days. The epidemic growth phase used to estimate the reproduction number consisted of 12 days for the greater Lima metropolitan area (May 31 to June 11) and 16 for the rest of Peru (June 10 to June 25). See Figure F in Text S1 for exact time periods considered as part of the epidemic growth phase.

### The impact of the winter school vacation on the age distribution of A/H1N1 influenza cases and the final pandemic attack rate

**Figure 5:**
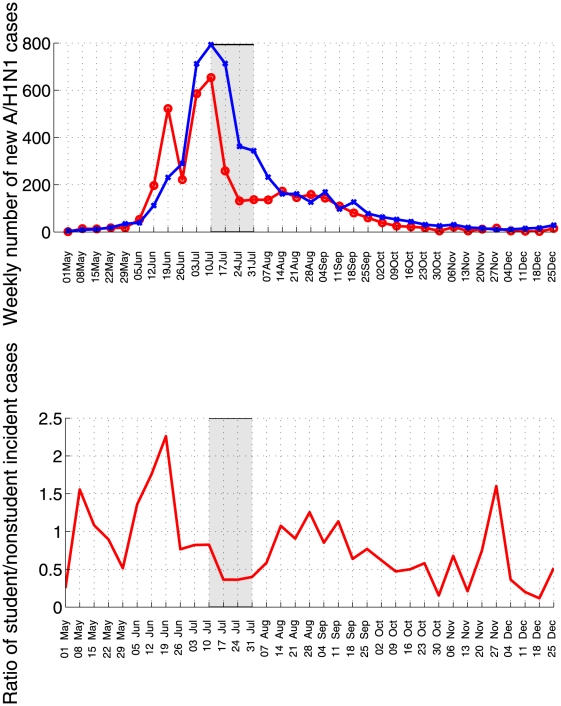
Weekly time series of A/A/H1N1 cases (top panel) among students (5–20 years, red curve) and all other age groups (blue curve). We also show the corresponding weekly ratio of student to nonstudent incident cases (bottom panel) across Peru, May 1, 2009 to December 31, 2009. The grey shaded area indicates the winter school vacation period (July16^th^ to August 6^th^).

**Figure 6:**
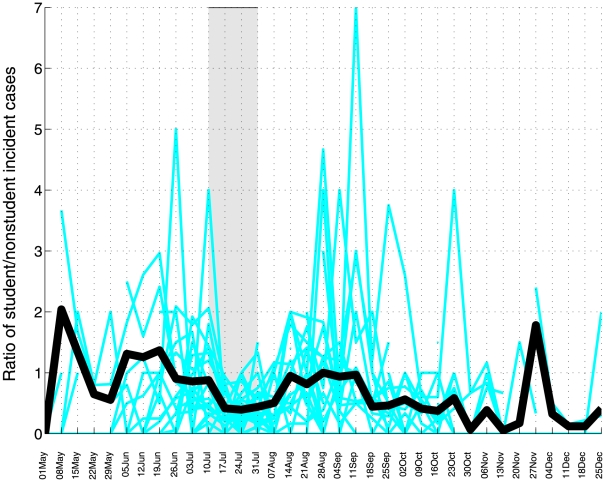
Weekly ratio of student to nonstudent incident cases of pandemic A/H1N1 influenza at the level of departments in Peru (light blue lines), May 01–December 31, 2009. The average trend in ratio of student to nonstudent cases across departments (thick solid black line) was weighted by the total number of confirmed A/H1N1 cases in each department. The grey shaded area indicates the winter school vacation period (July16^th^ to August 6^th^).

We analyzed the impact of the winter vacation period by monitoring temporal patterns in the ratio of incident student (defined as people 5–20 years of age) to non-student influenza A/H1N1 cases. At the national scale, this ratio was significantly below 1.0 during the winter school vacations and exceeded 1.0 two weeks following the resumption of school activities on August 6 (Wilcoxon test for differences in ratio of student to non-student cases before and during the winter school vacation period, P<0.001, Figure 5). At the level of departments, the average ratio of student to non-student influenza A/H1N1 cases decreased by 46% during the 3-week school closure period as compared to the preceding 6 weeks (T-test for differences in mean ratio, P = 0.007, Figure 6). Moreover, the ratio was lower and stayed below 1 for longer in the Lima metropolitan area than in the rest of Peru, consistent with the early pandemic wave changing the age structure of susceptible individuals in Lima in June 2009 (Figure G in Text S1).

Next, we explored the impact of the 2-week winter school break implemented after the peak of the pandemic on July 16, using an age-structured mathematical model of influenza transmission tailored to the population of Peru (Text S1). The predicted reduction in the final epidemic size obtained by reducing the transmission rate within the student population (i.e., <19 years) when R_0_ = 1.6 and R_0_ = 1.8 is shown in Figure H in Text S1 for various values of the percentage reduction of the transmission rate of the student population (20%–60%) and the timing of the start of the school closing period occurring a number of days after the epidemic peak. For an R_0_ of 1.6, a reduction of 30% in the transmission rate among the student population yields a 4–10% reduction in the pandemic attack rate when the 22-day school closing period takes place 5–20 days after the epidemic peak. A higher R_0_ value of 1.8 is associated with an even smaller reduction in the attack rate (4–7%). Overall we found that the reduction in the pandemic attack rate decreases significantly as R_0_ increases.

## Discussion

We have conducted a detailed analysis of the spatial-temporal characteristics of the A/H1N1 influenza pandemic in May–December 2009 at two levels of spatial aggregation, relying on a large sample of 18,139 laboratory confirmed cases and 22,994 ILI cases collected by a national epidemiological and virological surveillance system. We found substantial spatial variation in pandemic patterns across Peru, with two pandemic waves of A/H1N1 cases of varying timing and impact across geographic regions and age groups. The 2-week winter school vacation period in the second half of July 2009 was associated with changes in the age distribution of A/H1N1 cases, and earlier pandemic onset was correlated with large population size in the 24 departments. By contrast there was no association between pandemic dynamics and absolute humidity.

Our results suggest that pandemic virus activity began in Lima, consistent with the first case of pandemic A/H1N1 being detected in a Peruvian citizen returning to Lima from New York City on May 9th, 2009. We report a first wave of pandemic A/H1N1 transmission mostly focused on the Lima metropolitan area and peaking in late June, followed by a second wave affecting simultaneously coastal, mountain, and jungle regions of Peru and peaking in mid-July. It is likely that the movement patterns of infected tourists and traders disseminated the infection from the Lima metropolitan area to the rest of the country. A third increase in pandemic A/H1N1 case incidence was reported in the Jungle region in mid to late September 2009 and could be explained by lower population density and reduced connectivity in this region.

We found a significant reduction in the ratio of student to non-student cases during the winter school vacation period compared to the weeks before and after the winter break. Nevertheless, the winter school vacation period coincided with the downward phase of the pandemic in Peru. Our simulations based on a simple age-structured transmission model of influenza suggest that the winter school vacation period had a relatively small effect (∼10%) on reducing the overall burden of the pandemic. In contrast, school closure interventions implemented during the early phase of the 2009 A/H1N1 pandemic have been associated with a reduction in influenza transmission rate, estimated at 25% in Hong Kong [33] and 29–37% in Mexico [34].

While a previous study provided preliminary estimates of the reproduction number during the initial pandemic phase in Peru using an early series of confirmed A/H1N1 cases [35], our study is based on a consolidated dataset of the pandemic with high spatial and temporal resolution. Our R estimate for the first wave in the greater Lima metropolitan area was estimated in the range 1.7–2.2. Estimates of the reproduction number for the second wave concentrated in other regions of Peru were significantly lower than those obtained for the first wave in Lima (R∼1.3–1.5). The higher reproduction number of the first wave in Lima could be explained by the high contact rates characteristic of school age children, the most affected age group during this pandemic wave. Lower population density in other regions of Peru compared to the greater Lima metropolitan area could also support a lower reproduction number associated with the second pandemic wave. A recent review of reproduction number estimates for the 2009 A/H1N1 influenza pandemic in 20 countries reported a range of R between 1.1 and 3.1 with a median value of 1.6 [36]. R estimates have been reported in the range of 1.2–2.4 for community-based settings in Mexico [29], [34], [37], [38], Japan [39], New Zealand [40], Australia [41], Chile [42], Ontario, Canada [43], and the United States [44], while higher estimates ranging from 2.3 to 3.3 have been obtained during school outbreaks [6], [30], [45]. The variability in published estimates could be attributed to differences in estimation methods and assumptions, including different generation time distributions, inclusion of correction factors to adjust for case underreporting, and differences in the identification of the growth phase period [36]. Heterogeneity in the timing and intensity of intervention strategies, school activity periods, and climatic conditions [11], [24], [34] could have also contributed to differences in reproduction number estimates across locations.

We assessed the correlation of the timing of pandemic onset across spatial units as a function of population size, population density and absolute humidity levels. Our results indicate that areas with larger populations experienced earlier pandemic onset at the scale of the 24 Peruvian departments. This observation suggests a hierarchical dissemination pattern of the pandemic driven by large population centers of Peru, reminiscent of the 1918–1919 influenza pandemic in England and Wales [23] and seasonal influenza epidemics in the United States [46]. While absolute humidity has been found to be significantly associated with the onset of seasonal and pandemic influenza epidemics in the United States [11], [12], we did not find a significant correlation at the province level in Peru. Further analysis of the environmental or social factors influencing the transmission of seasonal and pandemic influenza is warranted in order to fully explain these patterns [47].

The first pandemic wave in Peru affected mostly school age children in the Lima metropolitan area. This pattern was not the result of testing or age population composition differences since testing rates were consistent across age groups in this region (Figure C in Text S1), and the population age distribution is very similar across geographic regions of Peru. The second major peak of cases affected all age groups and regions of Peru. Other countries experienced multiple pandemic waves including Mexico, the United States, and the United Kingdom, and Japan [45] [48], [49], [50] whereas a number of countries, particularly in the Southern Hemisphere, have experienced only a single pandemic wave in 2009, including Chile [51], Argentina [52], Australia [53], [54], and New Zealand [53]. Other countries in Europe also experienced a single main wave in fall 2009 [55].

Recent research has revealed that influenza circulation patterns differ across geographic regions in Peru in inter-pandemic periods [13], [19], [56]. Specifically, in the coastal region surrounding Lima, influenza circulates year round with particularly elevated numbers of cases in the winter time. In contrast, influenza transmission is focused in the cold season in the mountain region and is weakly seasonal in the jungle region where most transmission is limited to the rainy season during the first six months of the year [13], [19], [56]. The timing of the 2009 A/H1N1 influenza pandemic and contemporaneous winter influenza epidemics in Lima is in contrast with the timing of the 1918–1920 influenza pandemic in this city, which occurred during the summer periods of 1918–1919 and 1919–1920 [57].

There are several strengths and limitations in our study. First, one shortcoming of sentinel surveillance is the potential for sampling and selection bias, which preclude us from calculating reliable incidence rates in our study. Most of the 50 sentinel sites comprising our surveillance network covered populations across all age groups except for six sentinel sites that mostly captured adult populations. These sentinel sites were set up in two hospitals in Lima (Hospital 2 de Mayo and Hospital Edgardo Rebagliati Martins), three military hospitals in Lima, and one military health center in Trujillo (northwestern Peru). On the other hand, sentinel surveillance data can allow the identification of spatial-temporal variations in disease trends and of the viruses associated with those trends using fewer resources than required by a population-based study [13], [19]. A second caveat is related to the assumption that the initial growth rate estimated from Sentinel data closely tracks the “true” growth rate of the pandemic in the community, which we use to estimate the reproduction number. In our study >60% of all ILI cases were consistently tested for influenza in all regions throughout the main pandemic period. It is also reassuring that our R estimate for the initial wave was in close agreement with estimates obtained during the early pandemic phase in other countries [29], [37]. Third, the practice of testing changed on July 7 when the Peru Ministry of Health prioritized hospitalized cases for influenza testing in all health centers, except for the 50 original sentinel units. Nevertheless, the resulting decline in testing rates took place after the pandemic reached the peak across all regions of Peru, which eliminates the possibility that our estimates of the reproduction number and correlation analysis could be biased by changes in surveillance methodology.

In conclusion, our work suggests that the 2009 A/H1N1 influenza pandemic in Peru exhibited a rich spatial-temporal pattern with two pandemic waves of A/H1N1 cases of varying timing and impact across age groups and geographic regions. Larger population areas experienced earlier pandemic onset, suggesting a hierarchical influenza transmission pattern reminiscent of past influenza epidemics and pandemics. Overall, our findings suggest that population size, population density, and school activity periods can account for some of the observed variability in influenza pandemic patterns.

## Supporting Information

Text S1 Supplementary information.
(DOC)
