# Supplementary material for: Spatial and Temporal Characteristics of the 2009 A/H1N1 Influenza Pandemic in Peru
Source: PLoS One. 2011 Jun 21;6(6):e21287. doi: 10.1371/journal.pone.0021287 (PMC3119673; doi:10.1371/journal.pone.0021287)

**Spatial and temporal characteristics of the 2009 A/H1N1 influenza pandemic in Peru: Supplementary information**

Gerardo Chowell, Cécile Viboud, Cesar V. Munayco, Jorge Gómez, Lone Simonsen, Mark A. Miller, James Tamerius, Victor Fiestas, Eric S. Halsey, Victor A. Laguna-Torres

**Methods:** Influenza transmission model to quantify the relative impact of school closures

**References**

**Supplementary Figures A-H**

**Influenza transmission model to quantify the relative impact of school closures**

We used an age-structured transmission model to quantify the impact of school closing periods on the overall pandemic attack rate by reducing the transmission rate of the student population. A schematic of the compartmental epidemic model is shown below. A previous version of the model was developed to study the impact of vaccination strategies against pandemic influenza [1]. Briefly, each age group (indexed by *i*) is classified into 6 epidemiological states given by: susceptible (S_i_), latent (E_i_), symptomatic and infectious in the community (I_i_), hospitalized (H_i_), protected (P_i_), and dead (D_i_). Susceptible individuals in age group *i* are exposed to the influenza virus at the force of infection where $\beta_{\text{ij}}(t)$is the transmission rate at time t between age groups *i* and *j* and the total population size is given by

.

Population size by age group was obtained from population size estimates for 2009 in Peru. The transmission rates $\beta_{\text{ij}}(t)$ are given by qc_ij_(t) where q is the transmission probability per contact, which is assumed to be constant across age groups and c_ij_(t) are the age- and time-specific contact rates which are modeled based on a study describing self-reported age-specific contact rates in European countries [2,3]. Overall, the contact rate matrix is highly assortative with higher mixing rates within each age group than between age groups[3]. We assumed that the contact rate remained constant prior to the start of the school closing period. Starting on July 26, 2009 the contact rate within the student population (<19 y) was reduced by a factor of p, and the transmission rate parameter for the student population was reset to its original value at the end of the school closing period and remained constant for the remaining duration of the pandemic wave. Latent individuals E_i_ progress to the infectious class I_i_ at the rate k (1/k is the mean latent period of ~2 days). Infectious individuals are hospitalized at the age-specific mean rates α_i_ and recover at the mean rate γ_1_. Hospitalized individuals either recover at the constant rate γ_2_ or die from influenza at the age-specific rate δ_i_. While the age-specific hospitalization rates are adjusted using estimates of the probability of hospitalization given clinical illness by age group, the recovery rate γ_2_ is assumed to be constant across age groups for simplicity (2-4 days). Recovered individuals are assumed to remain protected for the duration of the epidemic. Infected individuals die with an age-specific mortality rates as described below. We assume that the entire population is susceptible to the novel influenza pandemic virus.

The reduction in the transmission rate among the student population was explored in the range 20-60% and the timing of the start of the school closing intervention was modeled relative to the number of days after the epidemic peak.

The system of differential equations that describes our influenza transmission model is given by

for $i=1,K,15$.

The hospitalization and mortality rates are given by α_i_ = ($p_{H_{i}}$/(1 − $p_{H_{i}}$))γ_1_ and δ_i_ = (*CFP*_i_/(1−*CFP*_i_))γ_2_, respectively, where $p_{H_{i}}$ and *CFP*_i_ denote the probability of hospitalization given clinical illness and the probability of death following hospitalization for age group *i* were based on estimates of the 2009 A/H1N1 influenza pandemic [1]. The system of ordinary differential equations was solved numerically using Matlab (The Mathworks, Inc) with initially 10 infectious young individuals (10-14 y).

The basic reproduction number, R_o_, measures the average number of secondary cases generated by a primary infectious in a completely susceptible population[4,5]. We set R_o_ in the range 1.6-2.6 by scaling the probability of transmission given contact (q) to a obtain a desired value of R_o_[1].

Schematic of the compartmental epidemic model for influenza transmission in Peru:

**
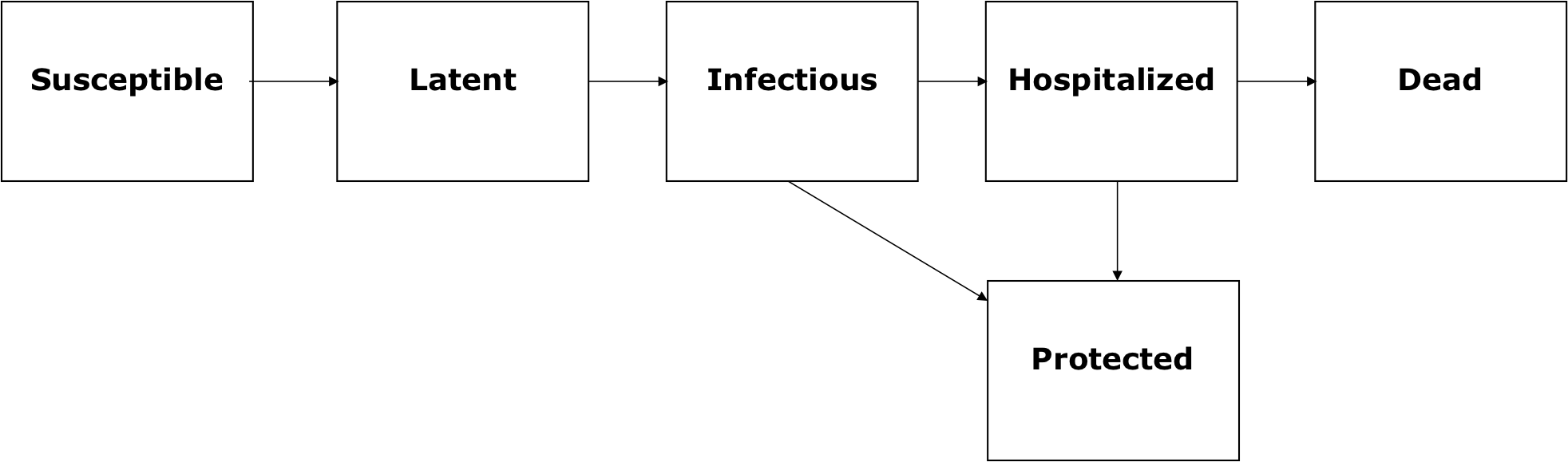
**

**References:**

1. Chowell G, Viboud C, Wang X, Bertozzi SM, Miller MA (2009) Adaptive vaccination strategies to mitigate pandemic influenza: Mexico as a case study. PLoS One 4: e8164.

2. Wallinga J, Teunis P, Kretzschmar M (2006) Using data on social contacts to estimate age-specific transmission parameters for respiratory-spread infectious agents. Am J Epidemiol 164: 936-944.

3. Mossong J, Hens N, Jit M, Beutels P, Auranen K, et al. (2008) Social contacts and mixing patterns relevant to the spread of infectious diseases. PLoS Med 5: e74.

4. Anderson RM, May RM (1991) Infectious diseases of humans. Oxford: Oxford University Press.

5. Diekmann O, Heesterbeek J (2000) Mathematical epidemiology of infectious diseases: model building, analysis and interpretation: Wiley.

**Supplementary Figures**

**Figure A**

Weekly variation in number of A/H1N1 cases (top) and in percent PCR positive (no. A/H1N1 positive cases/no. tests, bottom) in Lima and Callao and the rest of Peru, May 1 to December 31, 2009. The average PCR positivity was 42.8% (95% CI: 42.1, 43.5) in Lima and Callao and 51.6% (95% CI: 50.9, 52.3) in the rest of Peru.

**Figure B**

Weekly number of ILI cases (top) and weekly variation in testing rates (no. tests/no. ILI, bottom) in the greater Lima metropolitan area and the rest of Peru, May 1 to December 31, 2009. The average testing rate in Lima and Callao was 85.9% (95% CI: 83.5, 88.3) and 56.4% (95% CI: 51.2, 60.8) in the rest of the country (Wilcoxon test for differences in testing rates between geographic regions, P=0.03; Table S1).

**Figure C**

Age-specific variation in testing rates (no. tests/no. ILI) across coastal, mountain and jungle regions of Peru, May 1 to December 31, 2009. Overall, testing rates were consistent across age groups and geographic regions.


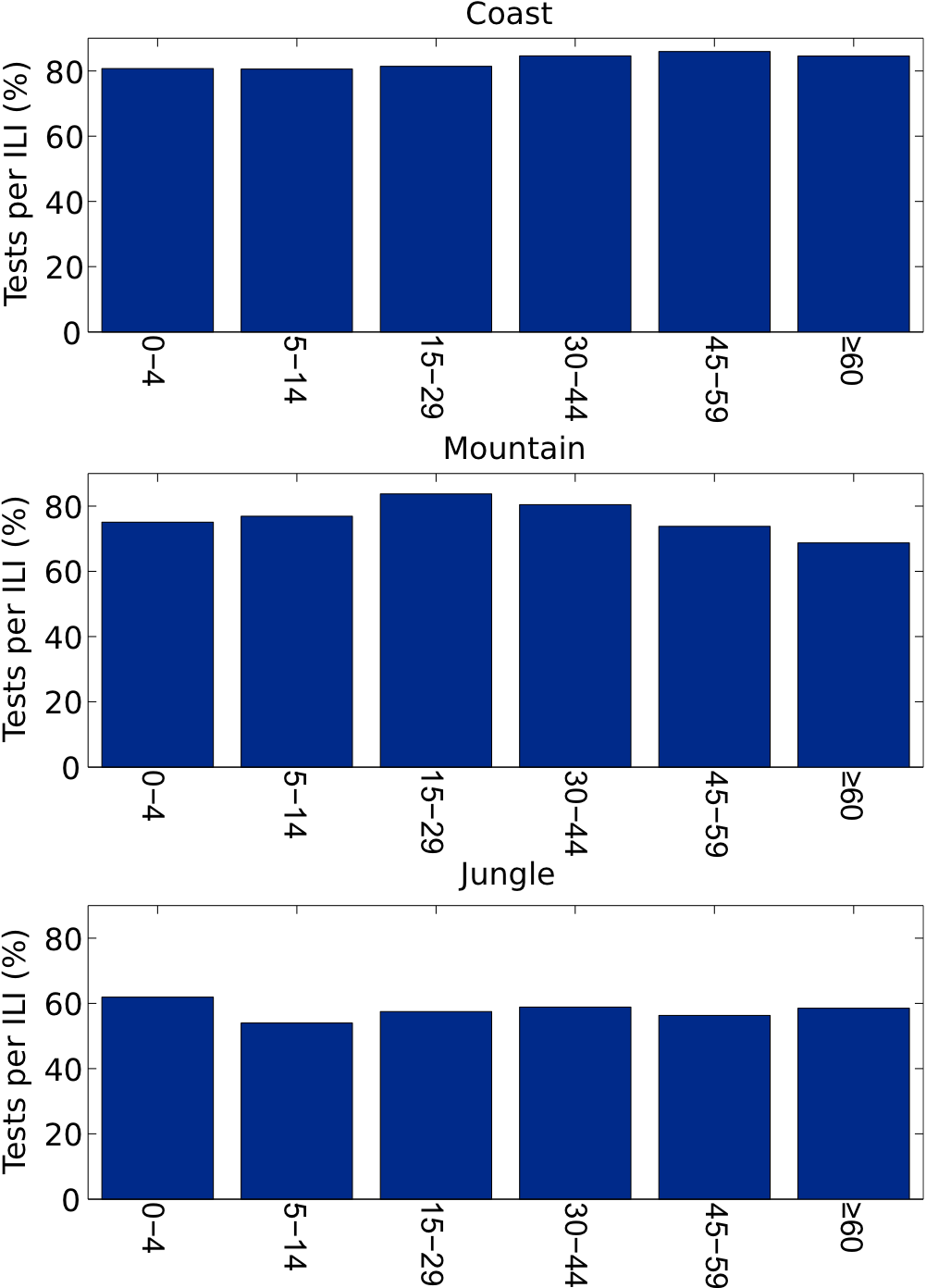


**Figure D**Timing of A/H1N1 pandemic onset (in days) as a function of population density across provinces. This suggests that larger population density provinces experienced earlier pandemic onset. The grey shaded area indicates the winter school vacation period (07/16 – 08/06).

**Figure E**

Daily variation in number of new A/H1N1 influenza cases by geographic region (top) and the average specific humidity by geographic region weighted by total number of A/H1N1 cases reported in each province, May 1 to December 31, 2009.


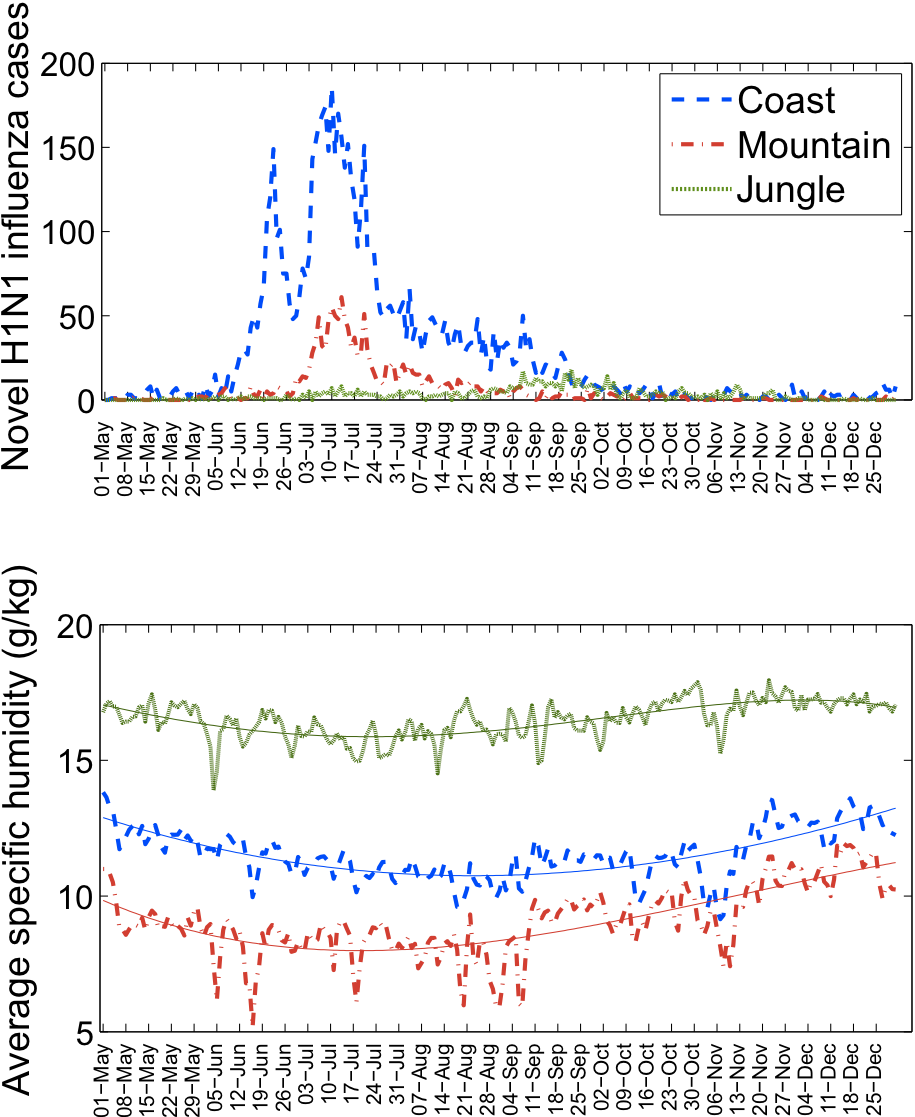


**Figure F**

Model fits (solid line) obtained after fitting an exponential curve to the growth phase of the pandemic onset phase (red dots) in two geographic regions of Peru to estimate the initial growth rate “r”. The growth phase that was selected to be representative of each of the pandemic waves consisted of 12 epidemic days for the pandemic onset in Lima and Callao and 16 days for the rest of Peru.

**Figure G**

The weekly ratio of student (5-20 years) to other age groups in the Lima metropolitan area (red o- curve) and the rest of Peru (blue –x curve). The grey shaded area indicates the winter school vacation period (07/16 – 08/06).


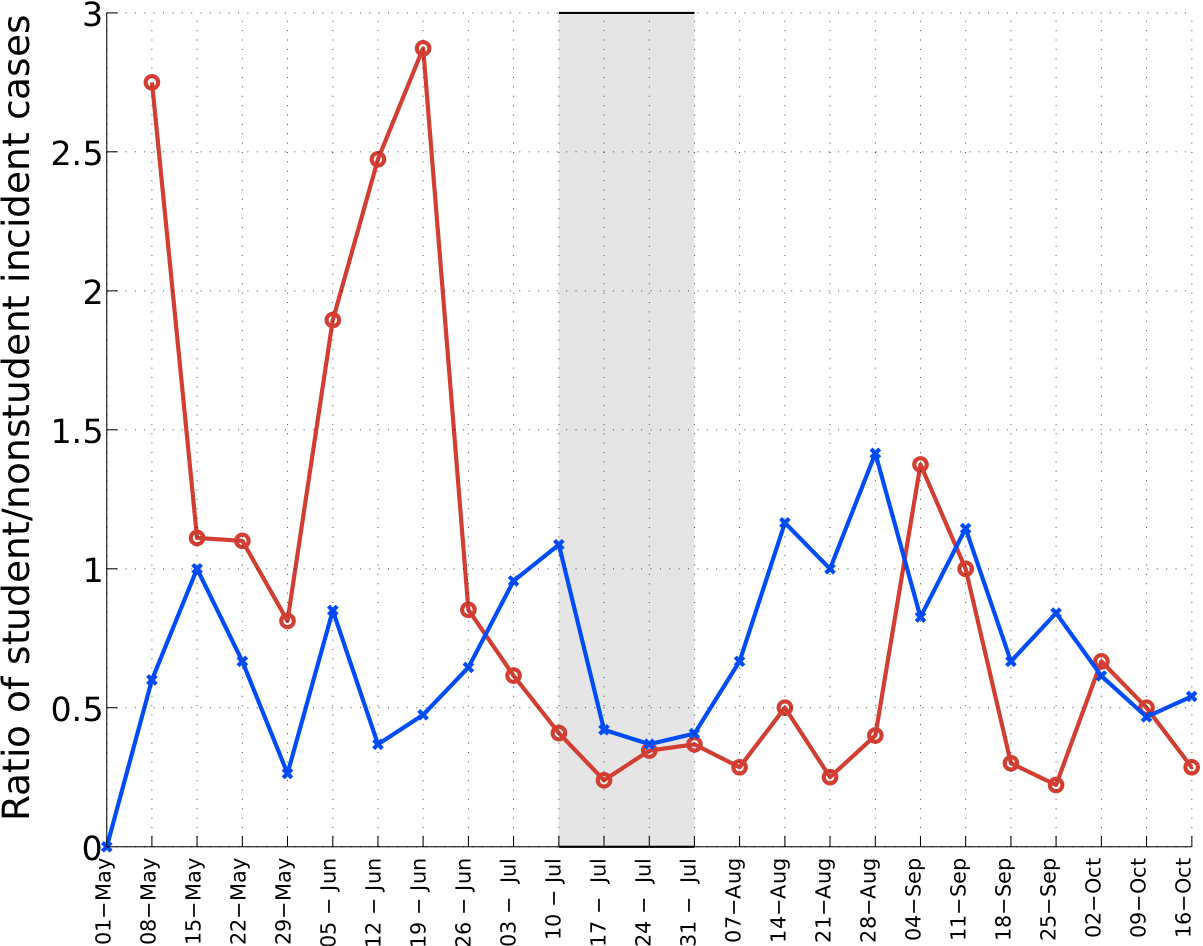


**Figure H**

Reduction in the final epidemic size (blue-red, %), as a function of the plausible reduction of the transmission rate among the student population (y-axis) and the timing of the start of a school closing period (x-axis) lasting for 22 days for two R_0_ values.


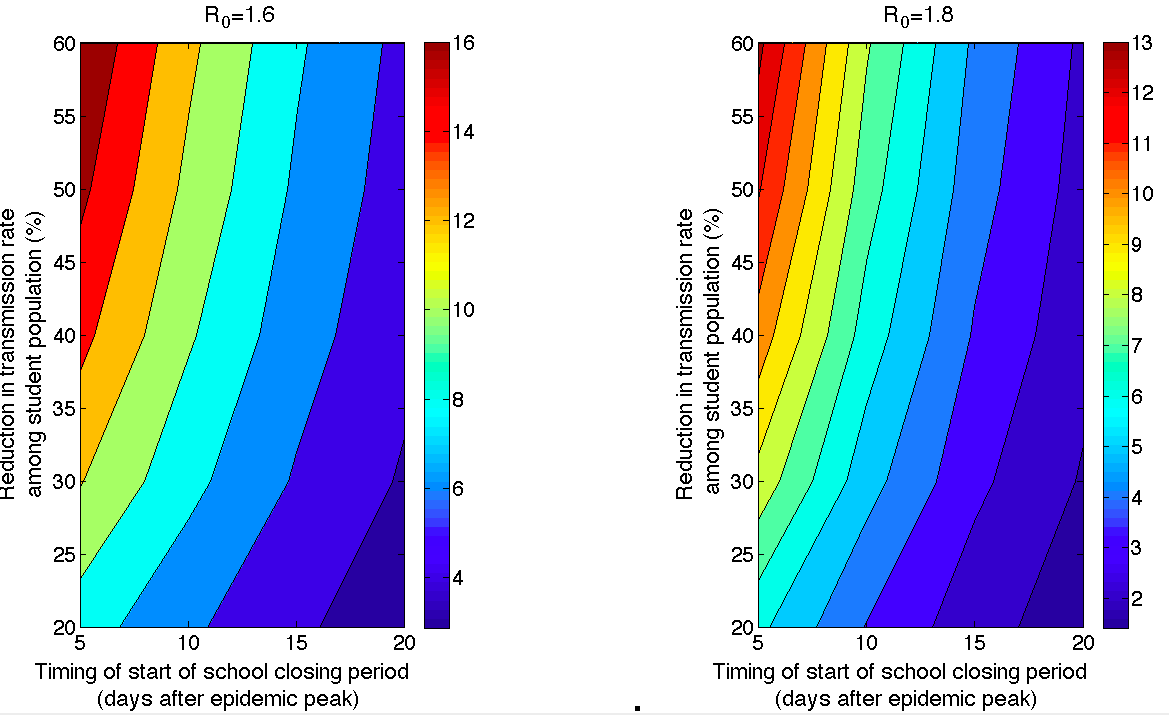

Supplement: Text S1 — Supplementary information. (DOC) [file pone.0021287.s001.doc]
